# Structural Basis for Broad Neutralization of Hepatitis C Virus Quasispecies

**DOI:** 10.1371/journal.pone.0026981

**Published:** 2011-10-26

**Authors:** Pascal Lapierre, Myriam Troesch, Fernando Alvarez, Hugo Soudeyns

**Affiliations:** 1 Service de Gastroentérologie, Hépatologie et Nutrition, Centre de Recherche du Centre Hospitalier Universitaire, Sainte-Justine, Montreal, Quebec, Canada; 2 Unité d'Iimmunopathologie Virale, Centre de Recherche du Centre Hospitalier Universitaire, Sainte-Justine, Montreal, Quebec, Canada; 3 Department of Microbiology and Immunology, Faculty of Medicine, Université de Montréal, Montreal, Quebec, Canada; 4 Department of Pediatrics, Faculty of Medicine, Université de Montréal, Montreal, Quebec, Canada; Institut Pasteur, France

## Abstract

Monoclonal antibodies directed against hepatitis C virus (HCV) E2 protein can neutralize cell-cultured HCV and pseudoparticles expressing envelopes derived from multiple HCV subtypes. For example, based on antibody blocking experiments and alanine scanning mutagenesis, it was proposed that the AR3B monoclonal antibody recognized a discontinuous conformational epitope comprised of amino acid residues 396–424, 436–447, and 523–540 of HCV E2 envelope protein. Intriguingly, one of these segments (436–447) overlapped with hypervariable region 3 (HVR3), a domain that exhibited significant intrahost and interhost genetic diversity. To reconcile these observations, amino-acid sequence variability was examined and homology-based structural modelling of E2 based on tick-borne encephalitis virus (TBEV) E protein was performed based on 413 HCV sequences derived from 18 subjects with chronic hepatitis C. Here we report that despite a high degree of amino-acid sequence variability, the three-dimensional structure of E2 is remarkably conserved, suggesting broad recognition of structural determinants rather than specific residues. Regions 396–424 and 523–540 were largely exposed and in close spatial proximity at the surface of E2. In contrast, region 436–447, which overlaps with HVR3, was >35 Å away, and estimates of buried surface were inconsistent with HVR3 being part of the AR3B binding interface. High-throughput structural analysis of HCV quasispecies could facilitate the development of novel vaccines that target conserved structural features of HCV envelope and elicit neutralizing antibody responses that are less vulnerable to viral escape.

## Introduction

Hepatitis C virus (HCV) is a blood-borne pathogen that chronically infects more than 125 million people worldwide [1]. Long-term HCV infection is associated with liver cirrhosis, hepatocellular carcinoma, and end-stage liver disease [2]. HCV is genetically diversified: it is classified into 6 major and >100 minor subtypes [3] and exists as a quasispecies within infected subjects [4], [5]. This high degree of genetic variability is thought to contribute to the persistence of HCV infections and to the pathogenesis of hepatitis C [6]. A large share of HCV sequence variation is concentrated within hypervariable regions of the E2 envelope gene, including hypervariable region 1 (HVR1), a sequence of 27 amino acids located at the N-terminus of E2 (amino acid residues 384–410) [7]. A second hypervariable cluster, termed HVR2, is located downstream from HVR1 (amino acid positions 474–482) [8], [9]. Finally, a third hypervariable region (HVR3) positioned in between HVR1 and HVR2 (amino acid residues 431–466) [10] was recently integrated in the canonical model of E2 structure [11]–[14]. Solvent exposure and the conservation of overall conformation and specific amino acid residues at specific positions of HVR1, HVR2, and HVR3 are consistent with roles in target cell recognition, virus attachment, and cell entry [10], [15]. As HCV E1 and E2 envelope glycoproteins are important targets for host humoral and cell-mediated immune responses, hypervariable regions are also subjected to robust levels of selective pressure (HVR1>>HVR3>HVR2) [10], [16], [17].

**Figure 1:**
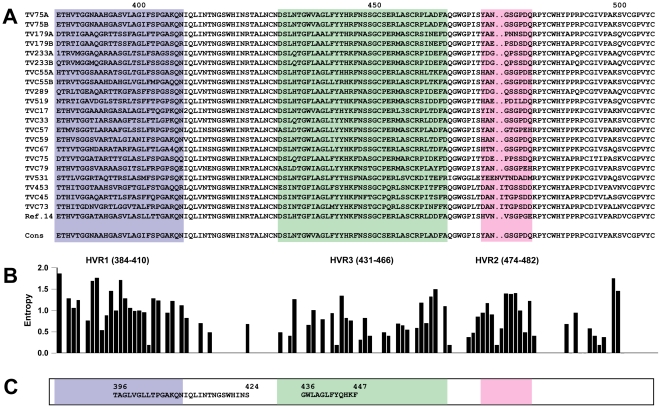
HCV E2 amino-acid sequence variability in HCV quasispecies derived from HCV-infected subjects. A. Consensus E2 amino-acid sequences were determined in 17 HCV-infected subjects and in the HCV-1a infected serum donor from ref. 14 based on the identity of the most frequent amino-acid residue at each position. 1: R or H; 2: V or I; 3: A or T; 4: R or Q. B. Variability at each amino acid position was computed using the Entropy-ONE Web tool [58]. C. Amino-acid segments that were shown to be important for binding of the AR3B antibody [14].

There is little evidence to link HCV-specific immunoglobulin (Ig) responses, spontaneous HCV clearance, and clinical progression of hepatitis C [18]–[20]. However, recently-published data based on cell-cultured HCV (HCVcc) and HCV pseudoparticles (HCVpp) indicate that broad antibody-mediated neutralization of HCV virions can in fact be achieved using human monoclonal antibodies (hMAbs) directed against epitopes located within HCV envelope proteins [21]. This and other reports [22]–[30] led to a shift in paradigm and have rekindled interest in HCV-specific neutralizing antibody responses. In some cases, HCV neutralization is thought to result from binding of E2 determinants that are critical for interaction with tetraspanin CD81 and/or scavenger receptor class B I (SR-BI) [22]–[25], two cell-surface molecules that are thought to be involved in attachment and entry of HCV into the host cell [31], [32]. Of particular interest, Law *et al.* reported that the AR3B hMAb was able to neutralize HCVcc and HCVpp expressing envelopes from multiple HCV subtypes and protect human liver-chimeric Alb-uPA/SCID mice against challenge with a heterologous HCV quasispecies [14], [33]. Based on antibody blocking experiments and alanine scanning mutagenesis, it was proposed that AR3B recognized a discontinuous conformational epitope comprised of E2 amino acid residues 396–424, 436–447, and 523–540. Intriguingly, one of these segments (436–447) overlaps with HVR3, a domain that exhibits significant intrahost and interhost amino-acid variability (Figure 1) [10]. To address the fundamental basis underlying the capacity of hMAbs such as AR3B to neutralize a heterogeneous quasispecies, E2 amino-acid sequence variability was examined and homology-based three-dimensional modelling of E2 based on tick-borne encephalitis virus (TBEV) E protein structure was performed using 413 HCV sequences derived from 18 subjects with chronic hepatitis C and 111 HCV sequences derived from reference sets. Here we report that regardless of a high degree of amino-acid sequence variability, the overall predicted structure of E2 was remarkably conserved, consistent with broad recognition of structural determinants rather than specific amino acid residues.

## Results

**Figure 2:**
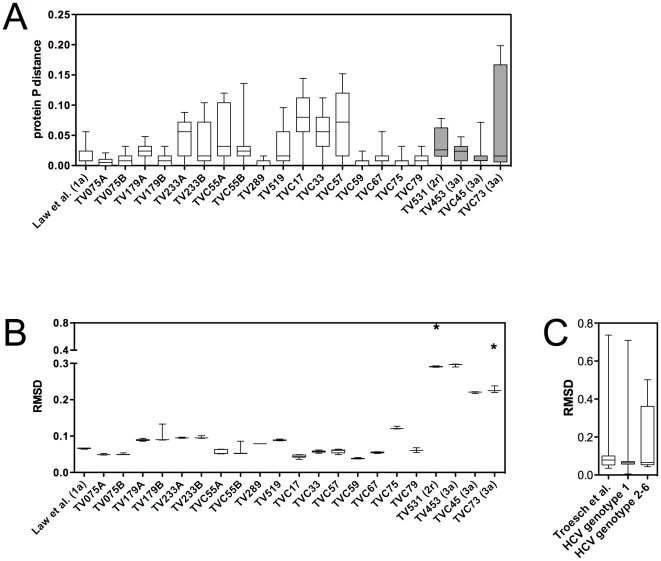
E2 amino-acid sequence variability and structural conservation across HCV subtypes and quasispecies. A. Pairwise protein p distances analysis in HCV-infected subjects revealed wide disparities in E2 (residues 384–508) amino-acid sequence variability. All patients were infected with HCV-1 except TV531, who was infected with HCV-2r, and TV453, TV45, and TV73, who were infected with HCV-3a (shaded bars). B. The majority of HCV-infected subjects showed minimal structural deviation from the E2 reference structure. Asterisks (*) indicate RMSD values associated with singular outlier structures that were observed in subjects TV531 and TVC73, but not in any other subjects. C. Modelled E2 structures from HCV-infected patients from our study group (n = 391) [10] and from the HCV Database Project (genotype 1; n = 90 sequences) [36] showed less than 1 Å deviation from the reference structure. Structures based on HCV genotypes 2–6 (n = 21 sequences) [36] also showed minimal structural variation. p: protein pairwise p distance. RMSD: root-mean-square deviation. Asterisks indicate RMSD values associated with these single outlying structures

HCV E2 amino-acid sequence variability was examined in HCV quasispecies derived from 17 HCV-infected subjects, 4 of whom were tested at two different time points (*i.e.* during the course of two consecutive pregnancies) (n = 21) (Figure 1) [10]. E2 sequences derived from the single HCV-1a-infected serum donor from the original report on AR3B-mediated HCV neutralization were also included in the analysis [14]. In the large majority of cases, pairwise protein p distances computed over a region of E2 that comprised the AR3B-defined neutralizing epitope (*i.e.* amino acid residues 384–508) indicated extensive amino-acid sequence variability (Figure 2A). Potential associations between protein p distances and clinical parameters measured among study subjects were examined. No statistically significant correlations were found between median pairwise p distances and: a) HCV viral load in terms log_10_ IU per ml plasma (p = 0.1033, r = 0.2950; Spearman's correlation test); b) circulating aspartate aminotransferase (AST) levels (p = 0.4419, r = 0.03599; Spearman's correlation test); or c) alanine aminotransferase (ALT) levels (p = 0.3974, r = −0.06395; Spearman's correlation test) (data not shown). Based on previous analysis [10] and data obtained in the present study, one plausible hypothesis to explain these findings would be that the dispersion in protein p distances reflects a manifestation of viral neutralization escape and selective pressure exerted on the viral quasispecies by the shifting of host HCV-specific immune responses in terms of antigenic specificity, scope, and magnitude. These could include neutralizing antibody responses directed against HCV E2 envelope protein and/or antibody-dependent cellular cytotoxicity (ADCC). In 9 of 21 cases (42.9%), median pairwise p distances were equal or significantly lower than that observed in the HCV-1a-infected serum donor (p<0.001, Kruskal-Wallis test with Dunn's post test). This suggests that AR3B-mediated neutralization of this particular HCV isolate in chimeric Alb-uPA/SCID mice, as reported by Law *et al.* [14], was not due to an unusual level of conservation of its quasispecies. Consistent with the fact that AR3B recognized HCV E2 in its native conformation and not under denaturing conditions, these observations further suggest that the specificity of AR3B could result from broad recognition of HCV E2 structural determinants rather than specific amino acid residues.

To test this hypothesis, homology-based structural modelling of E2 based on tick-borne encephalitis virus (TBEV) E protein (PDB ID 1SVB) was performed as previously described [10], [34], [35]. Separate structures were generated based on i) 391 different E2 amino acid sequences derived from 17 HCV-infected patients (see above) [10]; ii) 22 different E2 amino acid sequences derived from a serum donor infected with HCV-1a [14]; and iii) a set of 111 E2 reference sequences that comprised representatives of all 6 major HCV subtypes (http://hcv.lanl.gov/content/hcv-db/) [36]. The resulting 524 models were then individually compared to an E2 structure based on an HCV-1a reference sequence (GenBank accession no. M62321) using the secondary structure matching algorithm (SSM) [37]. Structural differences were expressed as root mean square deviations (RMSD), which represent the mean deviations in Å between paired protein backbones. Median RMSD values computed based on variants from the HCV-1a-infected serum donor were not significantly different from those obtained based on sequences from 14 of 21 (66.7%) HCV-infected sera tested [10] (p>0.05, Kruskal-Wallis test with Dunn's post test), indicative of extensive structural conservation throughout the proposed AR3B-binding domains in HCV-infected subjects (Figure 2B). Infection with HCV genotypes 2r (subject TV531; presence of a 2 amino acid insertion in E2) or 3a (subjects TV453, TVC45, and TVC73) explained 4 of 7 cases in which statistically significant structural differences were observed as compared with models based on E2 sequences from the HCV-1a-infected serum donor (Figure 2B). As above, potential associations between median RMSD and clinical parameters were examined among study subjects. There was no statistically significant correlations between median RMSD and: a) HCV viral load (p = 0.1675, r = 0.2274; Spearman's correlation test); b) circulating AST levels (p = 0.2856, r = 0.1387; Spearman's correlation test); or c) circulating ALT levels (p = 0.4472, r = −0.01408; Spearman's correlation test) (data not shown). Comparatively larger ranges in RMSD values were obtained when larger datasets were examined, including pooled sequences from the 17 HCV-infected subjects (n = 391) [10], HCV-1 reference sequences (n = 90) [36], and reference sequences from HCV subtypes 2–6 (n = 21) [35] (Figure 2C). However, median RMSD values were not significantly different between these 4 groups (p>0.05, Kruskal-Wallis test with Dunn's post test). It should be pointed out that although larger ranges of variance were found in the pooled samples, the differences are still of less than one Å (Figure 2). Interestingly, there was no correlation between median p distance and median RMSD among study subjects (p = 0.4989, r = −0.0006584; Spearman's correlation test) (data not shown). Therefore, E2 structural heterogeneity in individual HCV-infected subjects was not predicted by E2 amino acid sequence variability (Figure 2A and 2B).

**Figure 3:**
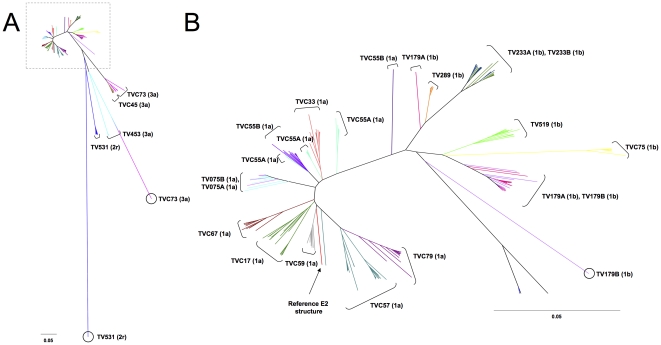
Dendrogram of E2 structure clustering in HCV quasispecies derived from HCV-infected subjects. A. Analysis of the structural distance (RMSD) matrix of modelled E2 structures from HCV-infected subjects (n = 391) using the neighbour-joining algorithm showed a clustering of genotype 1 variants (boxed), with the exception of singular outlier structures (circled) and genotype 3a variants. B. Subtype 1a and 1b clustered separately but were structurally similar. E2 structures derived from patients infected with the same HCV subtype formed distinct clusters.

**Figure 4:**
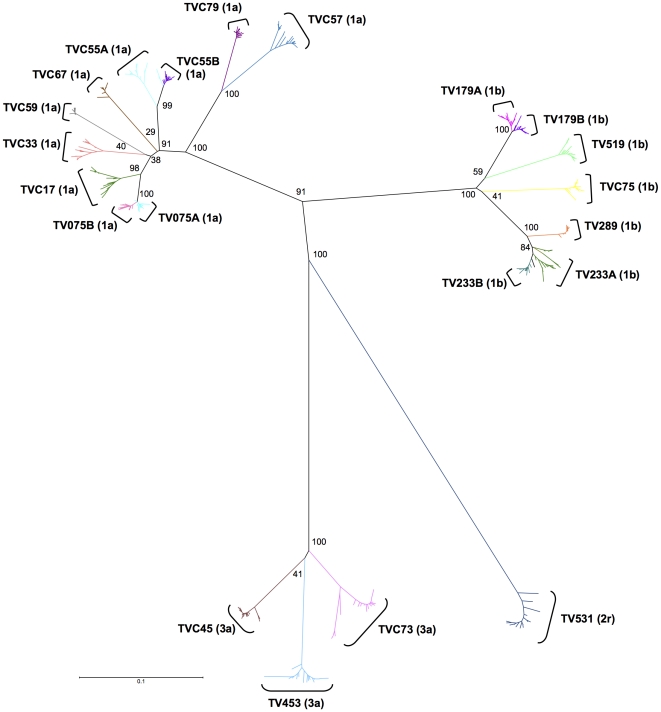
Phylogenetic analysis of HCV quasispecies derived from HCV-infected subjects. 391 independently-obtained HCV E2 nucleic acid sequences derived from HCV-infected subjects were analyzed using the neighbor-joining method, as described under Materials and Methods. 500 bootstrap re-samplings were performed to ascertain tree topology. Scale bar represents 0.1 nucleotide substitution per site.

Consequently, to understand the origin of E2 structural heterogeneity amongst quasispecies in these patients, E2 structural models (n = 391) and the E2 reference structure (GenBank accession no. M62321) were compared between each other and a RMSD distance matrix was computed. To better illustrate the relatedness of these multiple individual models, a dendrogram representing HCV E2 structure distribution was generated based on this matrix using the neighbor-joining method [38] (Figure 3). E2 structures from HCV genotype 1 formed a very tight cluster (0.13 Å diameter), indicative of significant structural similarity, while structures modelled based on sequences from subjects infected with HCV genotype 3a were more broadly distributed (0.73 Å diameter) (Figure 3A). In subjects TV531 (HCV-2r) and TVC73 (HCV-3a), singular outlier structures based on E2 sequences that exhibited insertions of two amino acid residues markedly diverged from structures modeled according to the sequence of other variants present in the same patients or sequences derived from other study subjects (Figure 3A). In contrast, when cladograms were computed based on nucleotide sequence data, these outliers invariably clustered with sequences from the same patients (bootstrap values >80%) (Figure 4). The apparent intrahost structural homogeneity of E2 and scarcity of outlier structures suggest that these could be the result of poorly-conserved mutations negatively affecting HCV replicative fitness. Alternatively, these outliers might represent newly emerged viral variants that escaped host humoral and/or cell-mediated immune responses [23], [29], [39]. E2 structures from subjects infected with genotype 1a or 1b clustered in a subtype-specific manner (Figure 3B). Subjects TV179 and TVC55 showed some divergent structures which could explain the larger range of similarities with the reference structure found in these patients (Figure 2B and 3B). These results suggest that E2 segments that comprise HVR3 exhibit a high degree of amino acid sequence variability while at the same time retaining a well-conserved structural framework, possibly related to their putative function(s) in HCV E2 conformation, E1–E2 dimerization, and/or viral entry into target cells [40].

**Figure 5:**
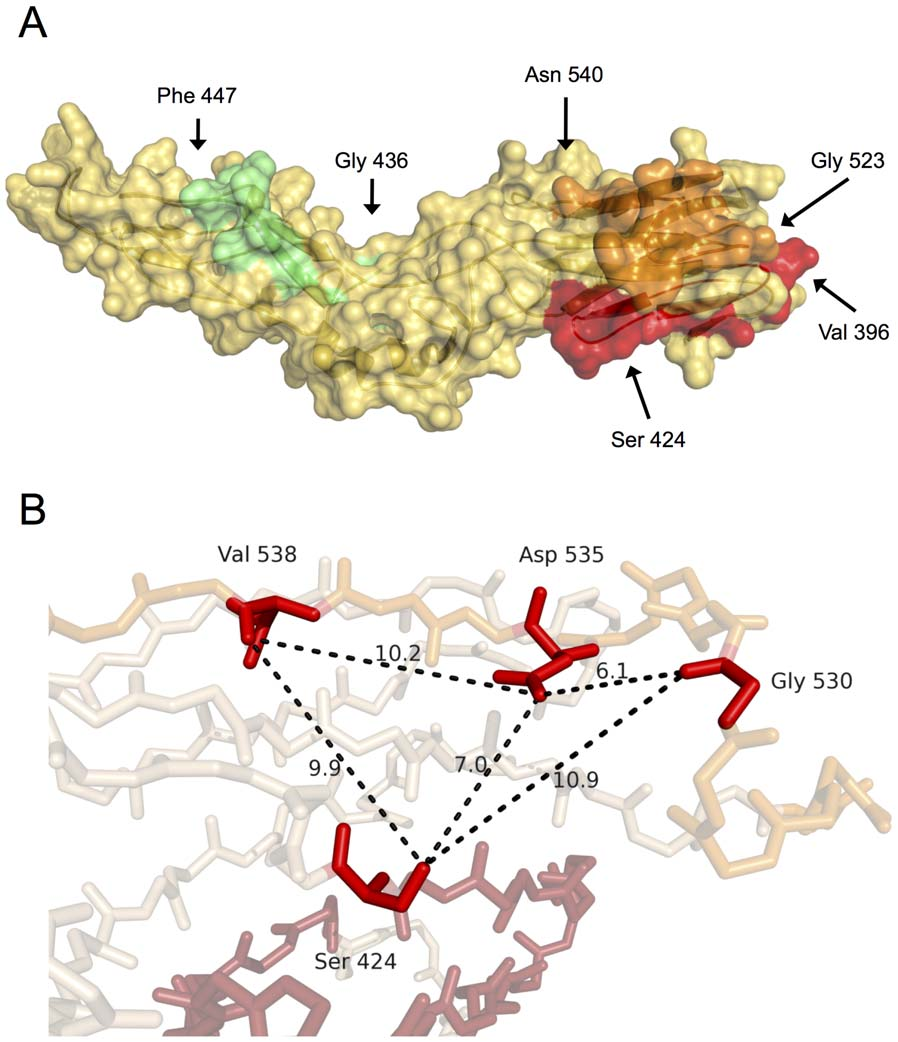
Putative binding site of the AR3B monoclonal antibody on E2. A. Structural analysis of the proposed AR3B binding site on E2 (orange, red, and green) revealed that it would bury between 1092 and 1794 Å^2^ at the surface of E2. Regions 396–424 (red) and 523–540 (orange) are closely associated compared with region 436–447 (green). B. Analysis of the 396–424 and 523–540 regions (magnified) showed that critical residues involved in AR3B binding (Ser 424, Gly 530, Asp 535 and Val 538) were largely surface-exposed and lied in close proximity to one another.

Since the effectiveness of AR3B in neutralizing HCV quasispecies may depend on its capacity to target conserved structural patterns, molecular modeling was used to examine the putative AR3B binding site on E2. Confirming antigenicity and accessibility calculations [10], segments that comprised amino-acid positions 396–424, 436–447, and 523–540 were predicted to be largely exposed at the surface of E2. Interestingly, regions 396–424 and 523–540 were predicted to lie in close spatial proximity and several amino acids that were identified in mutagenesis and antibody-blocking experiments as crucial residues for AR3-specific antibody recognition (*i.e.* Gly 530, Asp 535, and Val 538) were grouped within a 10.9 Å radius around Ser 424 (Figure 5). In sharp contrast, region 436–447, which overlaps with HVR3, is more than 35 Å away. According to this model, simultaneous binding of segments 396–424, 436–447, and 523–540 by a single immunoglobulin molecule would involve an interface area covering more than 1000 Å^2^ (Figure 5). In contrast, typical values for buried surfaces in antibody-protein interactions range between 560 Å^2^ and 855 Å^2^ [41]. Therefore, it is unlikely that the AR3B binding site on E2 encompasses HVR3 as previously suggested [14]. Instead, it is possible that binding of HVR3 by the 2/69A and 11/20 monoclonal antibodies but not 1/39 and 7/16B, all four of which map between amino acid residues 432 and 447 [14], induced conformational changes in E2 that inhibited AR3B interaction with its distal cognate binding sites.

## Discussion

In the present study, E2 sequences and structures from 524 HCV variants were examined using a secondary-structure graph-matching algorithm and displayed using a novel dendrogram-based graphical representation, allowing the rapid and precise comparison of hundreds of tri-dimensional structures. As similarities between large numbers of proteins could be assessed in an effort to identify common structural motifs, this high-throughput approach was particularly useful for the inspection of HCV quasispecies resulting from the high replication rate of HCV and the lack of proofreading activity of its RNA-dependent RNA polymerase. Overall, this analysis revealed that the primary amino-acid sequence of E2 was extremely diversified. This is consistent with the fact that E2 is exposed to significant selective pressure *in vivo*, and that continual amino acid sequence variation is associated with rounds of escape from host humoral and cell-mediated immune responses [42]. In contrast, the tridimensional structure of E2, including that of HVR1, HVR2, and HVR3, was largely maintained and showed little variation, except in the case of a limited number of outlier variants. In addition, estimates of buried surface were inconsistent with HVR3 being part of the AR3B binding interface. From these data, we propose that the broad recognition of E2 by MAb such as AR3B could result from their ability to target conserved structural conformations shared by multiple HCV variants rather than highly variable linear epitopes. Alternatively, AR3B neutralizing activity could be related to the prevention of a conformational change in the envelope protein that would be required for exposing a putative fusion peptide. Indeed, according to a recently-proposed model of E2 structure based on the conservation and positioning of intramolecular disulfide bonds [43], HVR3 (431–466) would straddle the « central » domain (DI) of E2, that participates in binding to CD81, and domain DII which is thought to comprise the putative fusion loop. In addition, based on testing of genotypic incompatibility in intersubtype chimeras, a portion of HVR3 (384–444) was proposed to play a role in the proper folding of DI [44]. Finally, in other *Flaviviridae* such as Dengue virus and TBEV, the native E protein is found as a dimer arranged in a « herringbone pattern » at the surface of the virion [45], [46]. Although we were unable to reliably model the putative dimer interface, an intriguing possibility would be that the conformational epitope recognized by AR3B actually spans both protomers in the native E2 dimer, leading to HCV neutralisation.

In humans, the majority of antibodies target discontinuous or conformational epitopes. Yet, most studies on B cell responses in HCV-infected patients have focused on the characterization of linear epitopes [47]. Characterization of antibodies with broad neutralization capacities and their conformational epitopes could lead to a better understanding of the fundamental basis for broad recognition of viral proteins with high amino-acid variability. Such broadly-neutralizing MAbs could be used as prophylaxis to prevent re-infection of the incoming organ in subjects undergoing liver transplant as a consequence of chronic hepatitis C [48]. While technically challenging, studies on conserved structural features of HCV envelope proteins could lead to the design of peptide-based structural mimics [49], which could in turn be used to elicit neutralizing–and potentially protective–antibody responses that are less vulnerable to mutational escape.

## Materials and Methods

### Ethics statement

This research protocol was conducted in full compliance with the Declaration of Helsinki and was approved by « le Comité d'éthique de la recherche du CHU Sainte-Justine », Montreal, Canada, where the study was conducted. Written informed consent was obtained from all study participants. All subjects and their children were provided with medical care and counselling required by their condition.

### Study subjects and clinical parameters

Study subjects (n = 17) were participants to the Centre maternel et infantile sur le SIDA mother-child cohort (CHU Sainte-Justine, Montreal, Canada) and were previously enrolled in a study of HCV-specific immune responses and HCV quasispecies evolution during pregnancy [10], [50]. Serum was extracted from whole blood by centrifugation and was kept at −80°C until used. ALT and AST levels were measured on a Synchron LX20 system (Beckman Coulter). Normal ALT and AST levels were 5–34 U/l and 11–43 U/l, respectively. Plasma HCV RNA levels were quantified using the COBAS Amplicor HCV Monitor assay version 2.0 (Roche Diagnostics). HCV genotyping was performed by sequence analysis of the 5′ non-coding region and NS5B, as described [51]. From these patients, 391 nucleotide sequences of E2 (mean of 23 sequences per subject; range = 17–38) were obtained and used for the analysis of amino acid variability, as previously described (GenBank accession no. DQ650805–DQ652141) [10]. Briefly, viral RNA was extracted from serum, and a portion of the E1 and E2 genes from HCV genotype 1 (nucleotide positions 1278–1889) was amplified by RT-PCR using the OneStep RT-PCR method (QIAGEN) and previously-published amplification conditions [10]. Primers E2/NS1a and E2/NS1b [52] were used to amplify HCV-1a and HCV-1b, while primers E2/NS5aBIS [10] and E2/NS1b were used for subject TVC55, and primers E2/NS3a and E2/NS1b or E2/NS3b [10] were used for subjects infected with HCV-3a. Because the mutation rate associated with the use of a non-proofreading polymerase is known to be largely unbiased with respect to the localization of mutations within amplified segments, comparisons between regions located within single amplicons were considered valid [53]. PCR products were purified from agarose gels and cloned in pCR2.1-TOPO (Invitrogen). DNA sequencing was performed on a Genetic Analyser 3100 (Applied Biosystems) using dye terminator chemistry. Chromatograms were edited manually using Chromas version 1.45 (Technelysium). Pairwise protein p distances were calculated using MEGA version 4.0 [54].

### Structural analysis of E2

Homology-based structural modelling of E2 based on TBEV E protein was performed as previously described [10]. Five hundred and twenty four separate structures were generated based on i) 391 different E2 amino acid sequences derived from 17 HCV-infected patients [10]; ii) 22 different E2 amino acid sequences derived from the HCV-1a serum donor described in reference [14]; and iii) a group of 111 E2 reference sequences that comprised representatives of all 6 major HCV subtypes [36]. Secondary structure predictions were used to align HCV E2 sequences with TBEV E protein, and the alignment was used to model E2 using E protein structure (PDB ID 1SVB) [34], [35] using MODELLER [55]. Energy minimization was performed using 100 steps of the steepest descent algorithm and CVFF [56]. Protein structures were visualized with PyMOL V1.3 (http://www.pymol.org). Resulting models were then individually compared to an E2 structure based on an HCV-1a reference sequence (GenBank accession no. M62321) using the SSM algorithm [37]. This procedure matches graphs built on the protein's secondary-structure elements followed by an iterative three-dimensional alignment of protein backbone cα atoms based on geometrical position rather than biochemical properties, allowing a precise assessment of proteins structural similarities in three dimension [37]. The 391 E2 structures from HCV-infected patients and E2 reference structure were also compared among themselves using SSM, resulting in a 392 by 392 structural distance matrix. Structural differences were expressed as root mean square deviations (RMSD), which represent the mean deviation in Å between paired protein backbones. Analysis of the structural distance (RMSD) matrix was performed using the neighbour-joining method [38], as implemented in MEGA version 4.0 [54].

### Phylogenetic analysis

HCV E2 sequences derived from 17 HCV-infected subjects (n = 391) were aligned using ClustalX 2.0.11 [57]. Kimura 2-parameter distance matrices were assembled and phylogenetic reconstructions were built according to the neighbour-joining method using MEGA [38], [54]. 500 boostrap resampling were used to assess the robustness of tree topology, with values >80% considered significant.

### Statistical analysis

Differences between groups were tested using the Kruskal-Wallis test with Dunn's post test. Correlation coefficients were computed using Pearson's test. All statistical analysis was performed using GraphPad Prism version 4 (GraphPad Software).

## References

[1] Shepard CW, Finelli L, Alter MJ (2005). Global epidemiology of hepatitis C virus infection.. Lancet Infect Dis.

[2] No authors listed (2002). NIH Consensus Statement on Management of Hepatitis C: 2002.. NIH Consens State Sci Statements.

[3] Simmonds P, Bukh J, Combet C, Deleage G, Enomoto N (2005). Consensus proposals for a unified system of nomenclature of hepatitis C virus genotypes.. Hepatology.

[4] Steinhauser DA, Holland JJ (1987). Rapid evolution of RNA viruses.. Ann Rev Microbiol.

[5] Martell M, Esteban JI, Quer J, Genesca J, Weiner A (1992). Hepatitis C virus (HCV) circulates as a population of different but closely related genomes: quasispecies nature of HCV genome distribution.. J Virol.

[6] Bukh J, Miller RH, Purcell RH (1995). Genetic heterogeneity of Hepatitis C virus: quasispecies and genotypes.. Sem Liv Dis.

[7] Weiner AJ, Brauer MJ, Rosenblatt J, Richman KH, Tung J (1991). Variable and hypervariable domains are found in the regions of HCV corresponding to the flavivirus envelope and NS1 proteins and the pestivirus envelope glycoproteins.. Virology.

[8] Kato N, Ootsuyama Y, Tanaka T, Nakagawa M, Nakazawa T (1992). Marked sequence diversity in the putative envelope proteins of hepatitis C viruses.. Virus Res.

[9] Kato N, Ootsuyama Y, Ohkoshi S, Nakazawa T, Sekiya H (1992). Characterization of hypervariable regions in the putative envelope protein of hepatitis C virus.. Biochem Biophys Res Commun.

[10] Troesch M, Meunier I, Lapierre P, Alvarez F, Boucher M (2006). Study of a novel hypervariable region in hepatitis C virus (HCV) E2 envelope glycoprotein.. Virology.

[11] McCaffrey K, Boo I, Poumbourios P, Drummer HE (2007). Expression and characterization of a minimal hepatitis C virus glycoprotein E2 core domain that retains CD81 binding.. J Virol.

[12] Cuevas JM, Torres-Puente M, Jimenez-Hernandez N, Bracho MA, García-Robles I (2008). Refined analysis of genetic variability parameters in hepatitis C virus and the ability to predict antiviral treatment response.. J Viral Hepat.

[13] Torres-Puente M, Cuevas JM, Jimenez-Hernandez N, Bracho MA, Garcia-Robles I (2008). Using evolutionary tools to refine the new hypervariable region 3 within the envelope 2 protein of hepatitis C virus.. Infect Genet Evol.

[14] Law M, Maruyama T, Lewis J, Giang E, Tarr AW (2008). Broadly neutralizing antibodies protect against hepatitis C virus quasispecies challenge.. Nat Med.

[15] Penin F, Combet C, Germanidis G, Frainais PO, Deleage G (2001). Conservation of the conformation and positive charges of hepatitis C virus E2 envelope glycoprotein hypervariable region 1 points to a role in cell attachment.. J Virol.

[16] Weiner AJ, Geysen HM, Christopherson C, Hall JE, Mason TJ (1992). Evidence for immune selection of hepatitis C virus (HCV) putative envelope glycoprotein variants: potential role in chronic HCV infections.. Proc Natl Acad Sci U S A.

[17] Kato N, Sekiya H, Ootsuyama Y, Nakazawa T, Hijikata M (1993). Humoral immune response to hypervariable region 1 of the putative envelope glycoprotein (gp70) of hepatitis C virus.. J Virol.

[18] Farci P, Alter HJ, Govindarajan S, Wong DC, Engle R (1992). Lack of protective immunity against reinfection with hepatitis C virus.. Science.

[19] Beld M, Penning M, van Putten M, Lukashov V, van den Hoek A (1999). Quantitative antibody responses to structural (core) and nonstructural (NS3, NS4, and NS5) hepatitis C virus proteins among seroconverting injecting drug users: impact of epitope variation and relationship to detection of HCV RNA in blood.. Hepatology.

[20] Adams G, Kuntz S, Rabelais G, Bratcher D, Tamburro CH (1997). Natural recovery from acute hepatitis C virus infection by agammaglobulinemic twin children.. Pediatr Infect Dis J.

[21] Meunier JC, Russell RS, Goossens V, Priem S, Walter H (2008). Isolation and characterization of broadly neutralizing human monoclonal antibodies to the E1 glycoprotein of hepatitis C virus.. J Virol.

[22] Grove J, Nielsen S, Zhong J, Bassendine MF, Drummer HE (2008). Identification of a residue in hepatitis C virus E2 glycoprotein that determines scavenger receptor BI and CD81 receptor dependency and sensitivity to neutralizing antibodies.. J Virol.

[23] Gal-Tanamy M, Keck ZY, Yi M, McKeating JA, Patel AH (2008). In vitro selection of a neutralization-resistant hepatitis C virus escape mutant.. Proc Natl Acad Sci U S A.

[24] Keck ZY, Li TK, Xia J, Gal-Tanamy M, Olson O (2008). Definition of a conserved immunodominant domain on hepatitis C virus E2 glycoprotein by neutralizing human monoclonal antibodies.. J Virol.

[25] Keck ZY, Olson O, Gal-Tanamy M, Xia J, Patel AH (2008). A point mutation leading to hepatitis C virus escape from neutralization by a monoclonal antibody to a conserved conformational epitope.. J Virol.

[26] Flint M, Maidens C, Loomis-Price LD, Shotton C, Dubuisson J (1999). Characterization of hepatitis C virus E2 glycoprotein interaction with a putative cellular receptor, CD81.. J Virol.

[27] Von Hahn T, Yoon JC, Alter H, Rice CM, Rehermann B (2007). Hepatitis C virus continuously escapes from neutralizing antibody and T-cell responses during chronic infection in vivo.. Gastroenterol.

[28] Johansson DX, Voisset C, Tarr AW, Aung M, Ball JK (2007). Human combinatorial libraries yield rare antibodies that broadly neutralize hepatitis C virus.. Proc Natl Acad Sci U S A.

[29] Keck ZY, Li SH, Xia J, von Hahn T, Balfe P (2009). Mutations in HCV E2 located outside the CD81 binding sites lead to escape from broadly neutralizing antibodies but compromise virus infectivity.. J Virol.

[30] Keck ZY, Saha A, Xia J, Wang Y, Lau P (2011). Mapping a region of HCV E2 that is responsible for escape from neutralizing antibodies and a core CD81-binding region that does not tolerate neutralization escape mutations.. J Virol.

[31] Pileri P, Uematsu Y, Campagnoli S, Galli G, Falugi F (1998). Binding of hepatitis C virus to CD81.. Science.

[32] Scarselli E, Ansuini H, Cerino R, Roccasecca RM, Acali S (2002). The human scavenger receptor class B type I is a novel candidate receptor for the hepatitis C virus.. EMBO J.

[33] Mercer DF, Schiller DE, Elliott JF, Douglas DN, Hao C (2001). Hepatitis C virus replication in mice with chimeric human livers.. Nat Med.

[34] Yagnik AT, Lahm A, Meola A, Roccasecca RM, Ercole BB (2000). A model for the hepatitis C virus envelope glycoprotein E2.. Proteins.

[35] Rey FA, Heinz FX, Mandl C, Kunz C, Harrison SC (1995). The envelope glycoprotein from tick-borne encephalitis virus at 2 Å resolution.. Nature.

[36] Kuiken C, Yusim K, Boykin L, Richardson R (2005). The Los Alamos hepatitis C sequence database.. Bioinformatics.

[37] Krissinel E, Henrick K (2004). Secondary-structure matching (SSM), a new tool for fast protein structure alignment in three dimensions.. Acta Crystallogr D Biol Crystallogr.

[38] Saitou N, Nei M (1987). The neighbor-joining method: a new method for reconstructing phylogenetic trees.. Mol Biol Evol.

[39] Dazert E, Neumann-Haefelin C, Bressanelli S, Fitzmaurice K, Kort J (2009). Loss of viral fitness and cross-recognition by CD8+ T cells limit HCV escape from a protective HLA-B27-restricted human immune response.. J Clin Invest.

[40] Stamataki Z, Grove J, Balfe P, McKeating JA (2008). Hepatitis C virus entry and neutralization.. Clin Liver Dis.

[41] Davies DR, Cohen GH (1996). Interactions of protein antigens with antibodies.. Proc Natl Acad Sci U S A.

[42] Shoukry NH, Cawthon AG, Walker CM (2004). Cell-mediated immunity and the outcome of hepatitis C virus infection.. Ann Rev Immunol.

[43] Krey T, d'Alayer J, Kikuti CM, Saulnier A, Damier-Piolle L (2010). The disulfide bonds in glycoprotein E2 of hepatitis C virus reveal the tertiary organization of the molecule.. PLoS Pathog.

[44] Albecka A, Montserret R, Krey T, Tarr AW, Diesis E (2011). Identification of new functional regions in hepatitis C virus envelope glycoprotein E2.. J Virol.

[45] Kuhn RJ, Zhang W, Rossmann MG, Pletnev SV, Corver J (2002). Structure of dengue virus: implications for flavivirus organization, maturation, and fusion.. Cell.

[46] Kiermayr S, Stiasny K, Heinz FX (2009). Impact of quaternary organization on the antigenic structure of the tick-borne encephalitis virus envelope glycoprotein E.. J Virol.

[47] Marceau G, Lapierre P, Beland K, Soudeyns H, Alvarez F (2005). LKM1 autoantibodies in chronic hepatitis C infection: a case of molecular mimicry?. Hepatology.

[48] O'Leary JG, Lepe R, Davis GL (2008). Indications for liver transplantation.. Gastroenterology.

[49] Ofek G, Guenaga FJ, Schief WR, Skinner J, Baker D (2010). Elicitation of structure-specific antibodies by epitope scaffolds.. Proc Natl Acad Sci U S A.

[50] Troesch M, Jalbert E, Canobio S, Routy JP, Bernard N (2005). Characterization of humoral and cell-mediated immune responses directed against hepatitis C virus F protein in patients coinfected with hepatitis C virus and HIV-1.. AIDS.

[51] Murphy D, Willems B, Delage G (1994). Use of the 5′ noncoding region for genotyping hepatitis C virus.. J Infect Dis.

[52] Farci P, Shimoda A, Coiana A, Diaz G, Peddis G (2000). The outcome of acute hepatitis C predicted by the evolution of the viral quasispecies.. Science.

[53] Polyak SJ, Sullivan DG, Austin MA, Dai JY, Shunhart MC (2005). Comparison of amplification enzymes for hepatitis C virus quasispecies analysis.. Virol J.

[54] Tamura K, Dudley J, Nei M, Kumar S (2007). MEGA4: Molecular Evolutionary Genetics Analysis (MEGA) Software Version 4.0.. Mol Biol Evol.

[55] Sali A, Blundell TL (1993). Comparative protein modelling by satisfaction of spatial restraints.. J Mol Biol.

[56] Dauber-Osguthorpe P, Roberts VA, Osguthorpe DJ, Wolff J, Genest M (1988). Structure and energetics of ligand binding to proteins: Escherichia coli dihydrofolate reductase-trimethoprim, a drug-receptor system.. Proteins.

[57] Larkin MA, Blackshields G, Brown NP, Chenna R, McGettigan PA (2007). Clustal W and Clustal X version 2.0.. Bioinformatics.

[58] Korber BTM, Kunstman KJ, Patterson BK, Furtado M, McEvilly MM (1994). Genetic differences between blood- and brain-derived viral sequences from human immunodeficiency virus type 1-infected patients: evidence of conserved elements in the V3 region of the envelope protein of brain-derived sequences.. J Virol.

